# Structural mobility tunes signalling of the GluA1 AMPA glutamate receptor

**DOI:** 10.1038/s41586-023-06528-0

**Published:** 2023-09-13

**Authors:** Danyang Zhang, Josip Ivica, James M. Krieger, Hinze Ho, Keitaro Yamashita, Imogen Stockwell, Rozbeh Baradaran, Ondrej Cais, Ingo H. Greger

**Affiliations:** 1 Neurobiology Division, Medical Research Council (MRC) Laboratory of Molecular Biology, Cambridge, UK; 2 Biocomputing Unit, National Center of Biotechnology, CSIC, Madrid, Spain; 3 Structural Studies Division, Medical Research Council (MRC) Laboratory of Molecular Biology, Cambridge, UK; 4 Present Address: Department of Physiology, Development and Neuroscience, University of Cambridge, Cambridge, UK

**Keywords:** Structural biology, Cellular neuroscience

## Abstract

AMPA glutamate receptors (AMPARs), the primary mediators of excitatory neurotransmission in the brain, are either GluA2 subunit-containing and thus Ca^2+^-impermeable, or GluA2-lacking and Ca^2+^-permeable^1^. Despite their prominent expression throughout interneurons and glia, their role in long-term potentiation and their involvement in a range of neuropathologies^2^, structural information for GluA2-lacking receptors is currently absent. Here we determine and characterize cryo-electron microscopy structures of the GluA1 homotetramer, fully occupied with TARPγ3 auxiliary subunits (GluA1/γ3). The gating core of both resting and open-state GluA1/γ3 closely resembles GluA2-containing receptors. However, the sequence-diverse N-terminal domains (NTDs) give rise to a highly mobile assembly, enabling domain swapping and subunit re-alignments in the ligand-binding domain tier that are pronounced in desensitized states. These transitions underlie the unique kinetic properties of GluA1. A GluA2 mutant (F231A) increasing NTD dynamics phenocopies this behaviour, and exhibits reduced synaptic responses, reflecting the anchoring function of the AMPAR NTD at the synapse. Together, this work underscores how the subunit-diverse NTDs determine subunit arrangement, gating properties and ultimately synaptic signalling efficiency among AMPAR subtypes.

## Abstract (web summary)

Calcium-permeable GluA1 AMPA glutamate receptors are structurally and functionally distinct from the prototypical GluA2-containing AMPA receptors, impacting their role in signal transmission, synaptic plasticity and learning.

## Main

AMPA glutamate receptors (AMPARs) mediate rapid signalling throughout the brain, and orchestrate various forms of synaptic plasticity that underlie learning^3^. These cation channels assemble in various combinations from four core subunits, GluA1–4, with each contributing unique gating kinetics, trafficking and subsynaptic localization^1,3–5^. Together with an array of auxiliary subunits, these receptors define the highly diverse postsynaptic response kinetics of neural circuits^1,6^. Incorporation of the GluA2 subunit renders AMPARs Ca^2+^-impermeable (herein referred to as ‘A2-containing’)^1^, an assembly that predominates across excitatory synapses. Nevertheless, high-conductance, Ca^2+^-permeable GluA2-lacking AMPARs (‘A2-lacking’) exist^7^, with critical functions particularly in interneurons and glia^2^. The most prevalent subtype is the GluA1 homomer, whose unique desensitization kinetics and inward rectification will impact short-term synaptic plasticity^8,9^. GluA1 homomers are upregulated under both physiological and pathological conditions^2,10^; contrary to GluA2, GluA1 receptors require long-term potentiation (LTP) stimuli for translocation into and stabilization at synapses^4,5,11,12^, where their Ca^2+^ signal contributes to potentiation^10,13^. GluA1 germline deletion results in loss of the AMPAR reserve pool required for LTP^14^, aberrant hippocampal place fields^15^ and defective working memory^16^.

AMPARs exhibit a modular architecture with a two-layer extracellular region connected to the transmembrane ion channel (transmembrane domain, TMD). A symmetry mismatch between the channel and the extracellular region gives rise to two conformationally distinct subunit pairs, A/C and B/D, which contribute uniquely to gating^17^. The four-fold symmetrical channel is gated by the ligand-binding domains (LBDs), adopting a two-fold dimer-of-dimers arrangement, which extends into the distal N-terminal domain (NTD) tier. The LBD and NTD dimers undergo domain swapping between the tiers^17^, with currently poorly understood consequences. The ion channel is encircled by diverse auxiliary subunits, modulating receptor gating kinetics, pharmacology and ion flux, with the transmembrane AMPAR regulatory proteins (TARPs γ2, γ3, γ4, γ5, γ7, γ8) being the most abundant^1,6^.

Current AMPAR structures are all A2-containing. In these, GluA2 preferentially occupies the ‘inner’ B/D positions^18,19^, which dominate gate dilation and stabilize the canonical ‘Y’ shape of the receptor through a tetrameric interface between NTD dimers^17^. Here, we show that GluA1 homomers substantially deviate from this organization. NTD sequence diversity leaves GluA1 without the tetrameric NTD interface of A2-containing receptors, rendering the NTD dimers highly mobile. These structural properties enable subunit reorganization in the LBD tier, culminating in a wider conformational landscape and expanded gating spectrum that influences synaptic transmission.

## A GluA1 TARP γ3 complex

**Fig. 1:**
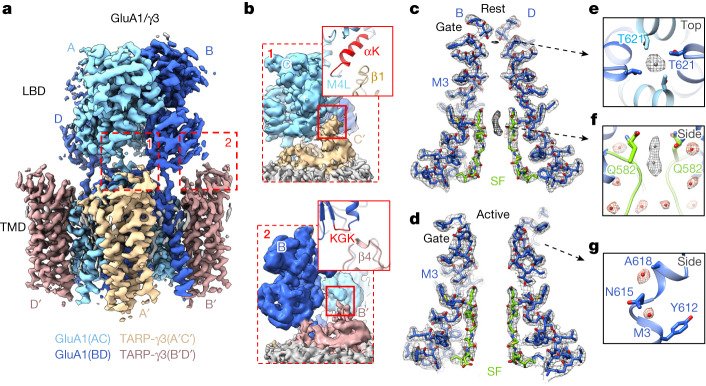
Organization of the active and resting GluA1/γ3 gating core. **a**, Resting GluA1/γ3 LBD–TMD map with GluA1 in two blue tones for subunit pairs A/C and B/D and γ3 in two browns for pairs A′/C′ and B′/D′. **b**, Magnified views of key TARP interacting elements on the LBDs (‘M4L’ denotes M4 linker). **c**, Conduction path of resting GluA1/γ3, showing M3 gating helices and SF (green) of B/D subunits; density at the gate and the SF are apparent. **d**, As in panel **c** but for the active state. **e**, Top view onto resting M3 gate, with an Fo-Fc density peak (contoured at 3.8*σ*), constituting a putative Na^+^ ion, coordinated by Thr621 side chains. **f**, Side view of the SF apex, where Gln582 side chains are coordinated by lateral waters (red) and permeating Na^+^ ions (grey; 3.8*σ*). Additional waters inside and outside the filter are shown beneath (red). **g**, Two waters localize to the kinked M3 helix of the B/D chains (4.85*σ*), next to Ala618.

**Extended Data Fig. 1:**
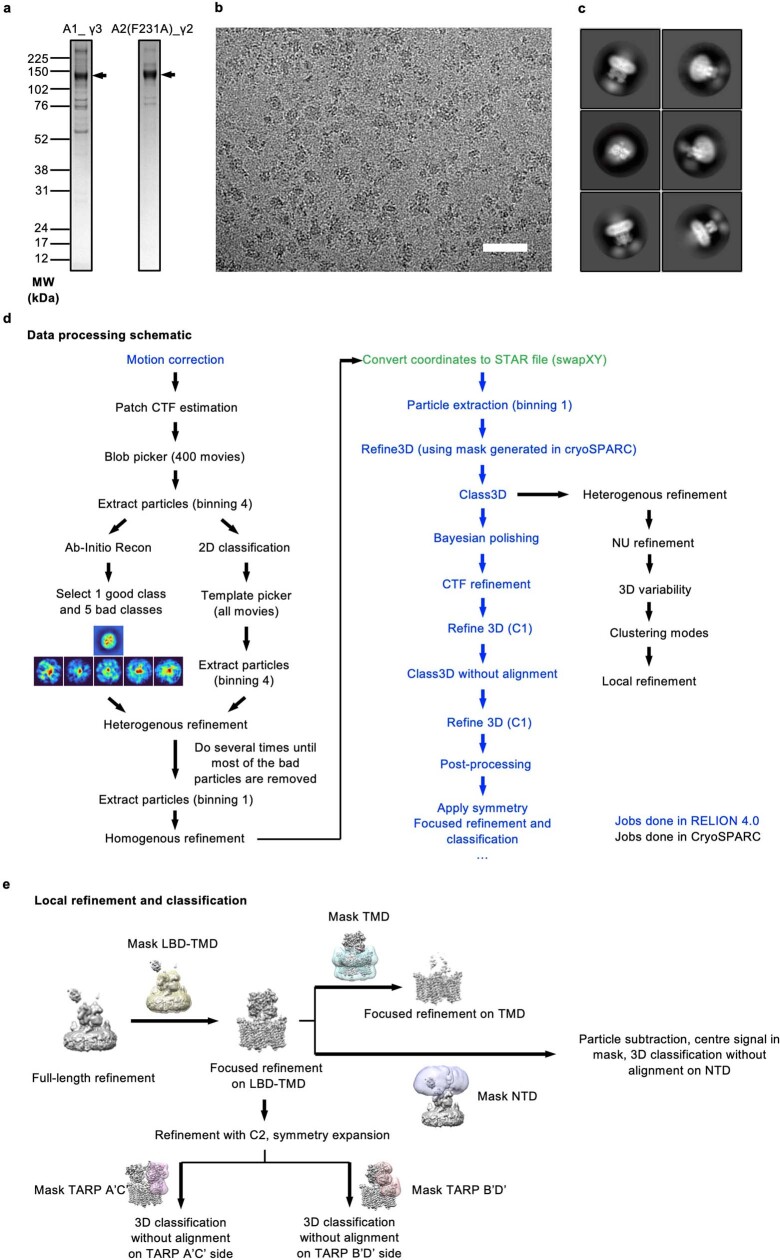
Purification of recombinant GluA1/γ3 and GluA2(F231A)/γ2 (Gly743) and representative Cryo-EM data processing workflow. **a**, Representative 4-12% Bis-Tris SDS-PAGE gel (of three purifications) stained with Coomassie blue, indicating elution of the GluA1/γ3 and GluA2(F231A)/γ2 (Gly743) complex from FLAG beads. For gel source data, see Supplementary Fig. 1. **b**, Representative motion-corrected micrograph of resting-state GluA1/γ3 (scale bar, 50 nm). **c**, Representative 2D class averages of resting state GluA1/γ3. **d**, General cryo-EM data processing workflow used for all data sets in this work. Raw movies were first motion-corrected in RELION (blue), and generated micrographs were then imported into CryoSPARC (black) for CTF estimation, particle picking, and cleaning. Selected particles were converted and imported back into RELION for Bayesian polishing, CTF refinement, and further 3D refinement. Refined or polished particles as appropriate were then returned to CryoSPARC for non-uniform refinement and 3D variability analysis. **e**, Focused refinement and classification scheme used for data sets in this work. Focused refinement on LBD-TMD or TMD regions was performed to improve the resolution. Classifications on TARP loops and NTD were performed by using soft masks on those regions or doing particle subtraction.

**Extended Data Fig. 2:**
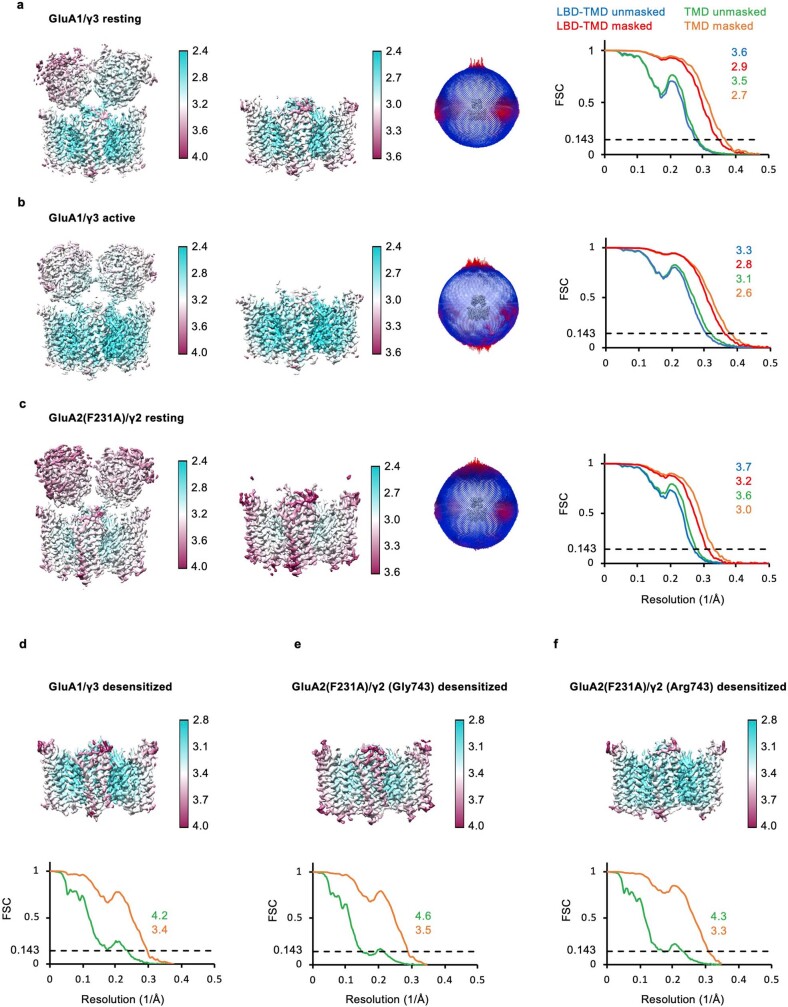
Cryo-EM analysis of GluA1/γ3, GluA2(F231A)/γ2 (Gly743) and GluA2(F231A)/γ2 (Arg743). **a**, Left: Local resolution maps for LBD-TMD and TMD of resting state GluA1/γ3, colored based on local resolution estimate as indicated by the color bar. Middle: Euler angle distribution of particles used for the cryo-EM reconstruction. Right: Masked (red, LBD-TMD and orange, TMD) or unmasked (blue, LBD-TMD and green, TMD) Fourier shell correlation (FSC) curves of corresponding maps with FSC = 0.143 (black line) used as a cutoff for corresponding resolution estimates shown. **b-c**, Local resolution maps, particle Euler angle distribution and FSC curves of open state GluA1/γ3 **(b)** and resting state GluA2(F231A)/γ2 (Gly743) **(c)** colored as in **a. d-f** local resolution maps (top) and FSC curves (bottom) for TMDs from desensitized GluA1/γ3 **(d)**, GluA2(F231A)/γ2 (Gly743) **(e)** and GluA2(F231A)/γ2 (Arg743) **(f)** colored as in **a**.

**Extended Data Fig. 3:**
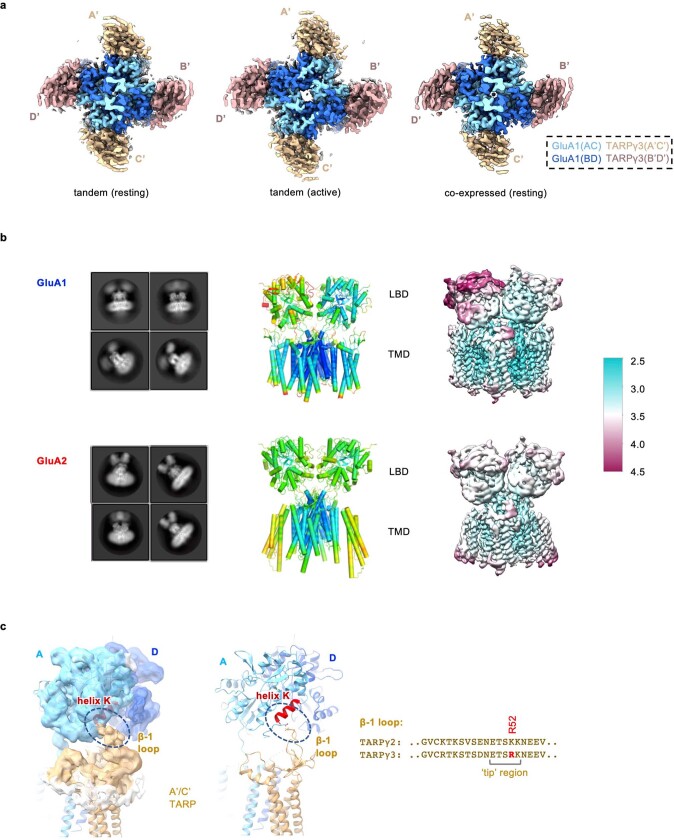
TARP interactions and mobility in resting state GluA1/γ3. **a**, Bottom views of GluA1/γ3 complex from tandem construct fusing GluA1 C-terminus with γ3 N-terminus (left) and co-expression of GluA1 with γ3 (right). **b**, 2D class averages, B-factor distributions and local resolution maps for GluA1/γ3 (top) and GluA1/2_γ8/CNIH-2 (PDB: 7OCE; ‘GluA2’; right). The GluA1/γ3 local resolution map is the resting one shown in Extended Data Fig. 2a, but represented differently for easier comparison. **c**, Key interactions of the TARP loops with the LBD are shown for an A’/C’ TARP (left and middle) with indicative interaction residues D765 in GluA1 chain A helix K and R52 in the TARP beta-1 loop shown as sticks and sequences of TARPγ2 and γ3 β1 loop (right). The putative region interacting with the LBD, around Arg52, is indicated by a square bracket (‘tip’).

To study a representative A2-lacking complex we focused on homomeric GluA1 associated with γ3, two AMPAR components enriched throughout the cortex^20^, which we trapped in resting, open and desensitized states (Methods and Extended Data Figs. 1 and 2). We either co-expressed GluA1 with γ3 or fused the GluA1 C terminus with the N terminus of γ3. Both approaches yielded a four-TARP stoichiometry, with γ3 occupying two pairs of non-equivalent binding sites termed A′/C′ and B′/D′ (Fig. 1a and Extended Data Fig. 3a), matching the behaviour of the closely related γ2 (ref. ^21^), but not γ8, which preferentially associates with the B′D′ sites^22^. Hence, we obtained a receptor saturated with four auxiliary subunits, mirroring neuronal AMPARs.

Our cryo-electron microscopy (cryo-EM) maps exhibited conformational heterogeneity, which remained following focused analysis on the LBD–TMD core. This contrasts with our structures of A2-containing receptors^18,22^ (Extended Data Fig. 3b), and implies greater flexibility of GluA1/γ3. We nevertheless obtained maps of the LBD–TMD sector with nominal overall resolution of sub-3 Å (Extended Data Table 1), and resolved large parts of the interaction between the TARP and the LBDs (Fig. 1a,b and Extended Data Fig. 3c). The desensitized state deviated even more with extensive rearrangements in the LBD tier, as discussed later. We first describe new features of the well-resolved TMD/TARP sector in resting and open states (Fig. 1a–d).

## The GluA1/γ3 ion conduction path

**Extended Data Fig. 4:**
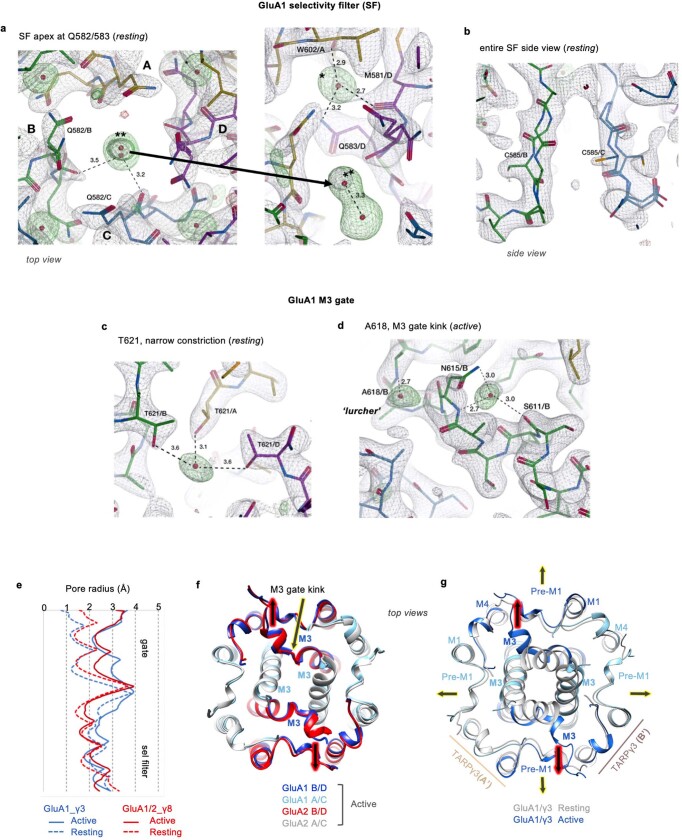
Features in the TMD of resting and open state GluA1/γ3. **a**, Peaks corresponding to sodium ions observed at the selectivity filter entry in the resting state from top (left) and bottom (right) views. The putative Na^+^ ion is marked with two asterisks. A strong water molecule locates to the periphery of Q583, connecting it with M581 and W602 in a neighboring subunit (this water is denoted with one asterisk). **b**, side view onto the selectivity filter showing the mobile C585 modelled in double occupancy. **c**, A putative Na^+^ ion locates to the narrow M3 gate constriction and is coordinated by the four T621 side chains, one from each subunit. **d**, Open state-specific water molecules that may stabilize the M3 kink, a hallmark of the AMPAR open state. Hydrogen bonds indicated by stippled lines, with distances in Ångstrom shown. All Fo-Fc difference peaks (green mesh) are contoured at 5.5σ. **e**, Pore dimensions of resting state (stippled lines) and active states (full lines) for GluA1/γ3 (blue) and GluA1/2 (red; PDB: 7OCF). **f**, Top view of superposed active state GluA1/γ3 and GluA1/2_γ8/CNIH2 (PDB: 7OCF) models, showing close similarity and gating dominance of the B/D chains (black arrows, red glow). **g**, Top view of superposed GluA1/γ3 models at resting (grey) and open state (colored) shows the asymmetric gate dilation as in B, which is accompanied by widening of the preM1 helices in the active versus resting state.

Improved resolution (approximately 2.5 Å), together with the calculation of Fo-Fc difference maps, reveals putative ions and water molecules concentrating at the GluA1 gate and the selectivity filter (SF), in a state-dependent manner (Fig. 1e–g). Water fills the cavities behind the flexible SF and coordinates the Gln582 side chains, which determine Ca^2+^ permeation (Fig. 1f and Extended Data Fig. 4a,b)^23^. These waters appear in both active and resting states, and together with the highly flexible Cys585 filter residue^22^ are poised to impact dynamics of the SF (Extended Data Fig. 4b) and, in turn, conductance. In the closed channel, putative Na^+^ ions, based on geometry and coordination chemistry, locate within the SF and are coordinated by Thr621, which forms the gate constriction (Fig. 1e,f and Extended Data Fig. 4c). These density peaks are not seen in the open state, despite the presence of 10 mM Ca^2+^ during protein purification, implying weaker ion binding in a conducting pore configuration^24^.

The GluA1 M3 gate exhibits the same gate asymmetry as A2-containing receptors, revealing that subunit position rather than identity determines activation gating in AMPAR subtypes: the critical M3 gating linkers of the GluA1 B/D subunits extend horizontally and their M3 helices kink to enable gate opening, whereas the A/C M3 helices only experience a subtle vertical deflection (Fig. 1d and Extended Data Fig. 4e–g). Water molecules locate to the M3 kink at the ‘lurcher’ residue Ala618 (Fig. 1g and Extended Data Fig. 4d), which may stabilize the B/D subunits in a kinked, open-gate conformation. The Na^+^ ion at Thr621, on the other hand, could favour a closed-gate state.

## TARPγ3 interaction with the LBD

We resolve various contacts between the flexible γ3 extracellular loops and the GluA1 LBD. These include different interactions between the A′C′ versus the B′D′ TARPs, enabling the diverse regulation of AMPAR properties^6^. Particularly noteworthy is the selective engagement of LBD helix K, next to the M4 gating linker, by the A′C′ TARP (via the β1-loop) (Fig. 1b and Extended Data Fig. 3c)^25^. These contacts are absent at the B′D′ sites, where the TARP β4-loop instead interacts with the LBD KGK-motif (Fig. 1b) to influence gating through currently unresolved mechanisms^26–28^. The inner TARP loop (Ex2) directly engages the critical M1 and M3 gating linkers at the A′C′ but not B′D′ sites. Together, these contact sites differ from those formed by γ8 (ref. ^24^), but are broadly in line with those seen with γ2 (ref. ^21^), highlighting binding site-specific AMPAR modulation by TARP subtypes^6^.

## GluA1 NTD dynamics

**Fig. 2:**
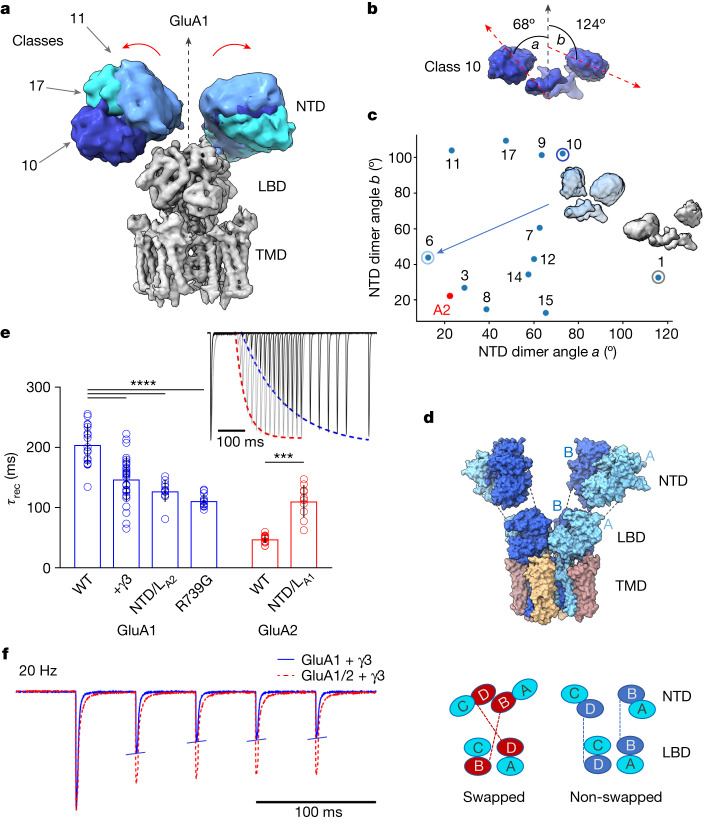
Structure and function of the GluA1/γ3 NTD tier. **a**, Representative NTD 3D class averages in different blues aligned to LBD and TMD of a full map (grey), illustrating NTD dynamics. **b**, Two splaying angles, *a* and *b*, between the local two-fold axes of fitted models for each NTD dimer and the global two-fold axis of a reference full-length receptor (PDB: 6QKZ), illustrated for representative class 10 (yellow in Extended Data Fig. 5a). **c**, Conformational landscape of fitted models with angles *a* and *b* along *x* and *y* axes, showing two extreme classes. A2 (red) denotes reference (PDB: 6QKZ). **d**, Fitted model of non-swapped GluA1/γ3 (top), with schematic showing the divergence of NTD and LBD placements from the canonical swapped conformation (bottom). **e**, Desensitization recovery for various GluA1 (blue) and GluA2 (red) constructs (mean ± s.d.; Welch’s analysis of variance (ANOVA) (*W*_5,33.88_ = 160.6; *P* < 0.0001) with Dunnett’s multiple comparisons; ***P = 0.0004, ****P < 0.0001; see Extended Data Table 2; number of cells: GluA1: 17 (WT), 31 (+ γ3), 11 (NTD/L_A2_), 13 (R739G); GluA2: 14 (WT), 10 (NTD/L_A1_)). Inset, representative paired-pulse recordings. Ratios of second and first peaks were plotted against time and fitted with a single exponential to obtain *τ*_rec_ (43 ms for red GluA2 unedited Arg and 192 ms for blue GluA1). **f**, Overlaid representative current traces from outside-out HEK293-cell patches elicited with 10 mM glutamate, 2 ms pulses at 20 Hz frequency, for GluA1 + γ3 (blue) and GluA1/2 + γ3 (red). WT, wild type. Source Data

**Fig. 3:**
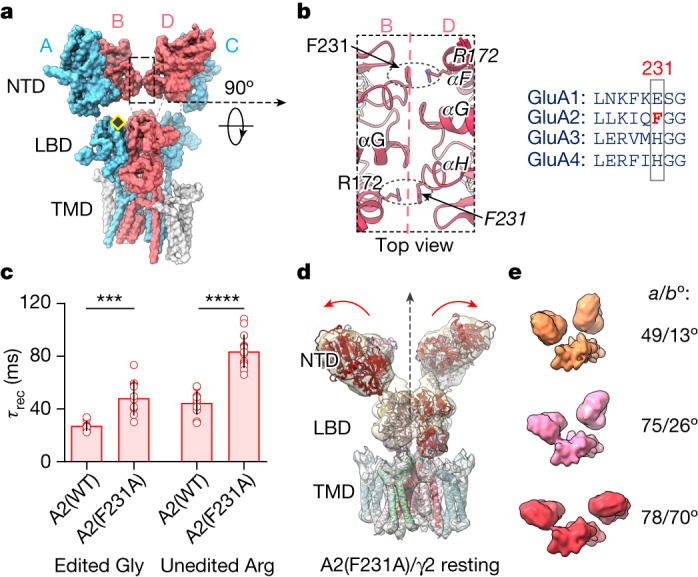
Role of the B/D NTD interface in GluA2/γ2. **a**, A2-containing receptor (PDB: 6QKZ), with GluA2 subunits in B/D position that form the NTD dimer-of-dimers interface (boxed) in red, GluA1 subunits in A/C position in blue and TARPs in grey. The yellow diamond indicates the R/G editing site. **b**, Left, magnified NTD dimer-of-dimers interface shows critical cation–pi interactions between Arg172 and mutated residue Phe231. Right, alignment shows mouse AMPAR paralogues surrounding Phe231. **c**, Summary data for desensitization recovery of GluA2 (unedited Arg and edited Gly), showing the effect of F231A, presented as mean ± s.d. Welch’s ANOVA (*W*_3,22.8_ = 81.31; *P* < 0.0001) followed post hoc with Dunnett’s multiple comparisons: ***P = 0.0009, ****P < 0.0001. Number of cells: edited Gly: 8 (A2(WT)), 12 (A2(F231A)); unedited Arg: 12 (A2(WT)), 14 (A2(F231A)). **d**, GluA2(F231A)/γ2 composite map, showing loss of NTD tetramer interface and dimer splaying (red arrows); vertical stippled arrow denotes global two-fold symmetry axis. **e**, Representative GluA2(F231A)/γ2 NTD classes showing variation similar to GluA1/γ3. Source Data

**Extended Data Fig. 5:**
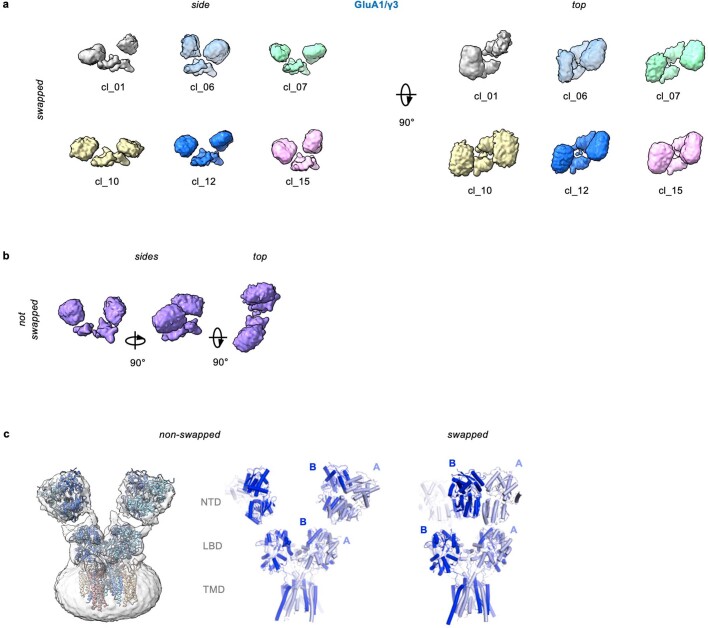
NTD flexibility in GluA1/γ3 resting state. **a**, Side (left) and top (right) views of six representative NTD classes from resting state GluA1/γ3 show separation and splaying of the NTD dimers. **b**, A 3D NTD class from non-swapped receptors, an NTD dimer is continuous with its respective LBD dimer. **c**, Cryo-EM map and fitted model of non-domain swapped GluA1/γ3 (left) and model alone (middle) of non-swapped GluA1/γ3 versus swapped GluA1/γ3 (right).

Substantial deviations from A2-containing receptors were apparent in the GluA1 NTD tier, the sequence-diverse upper half of an AMPAR^6^. The tetrameric B/D NTD interface common to GluA2 receptors is lost in GluA1, resulting in highly flexible NTD dimers of low resolution (Fig. 2a versus Fig. 3a), as already apparent in two-dimensional (2D) class averages (Extended Data Fig. 3b). A similar behaviour has been noted for desensitized GluA2 lacking auxiliary subunits^29,30^, and in native AMPARs^31^. Contrary to these, intact Y-shaped GluA1 was not apparent, even under resting-state conditions. Configurations of the GluA1 NTD dimers in three-dimensional (3D) classes range widely, from vertical upright to horizontally splayed, with some even bending into contact with the LBD (class_01) (Fig. 2b,c and Extended Data Fig. 5a). By fitting atomic models of NTD dimers (PDB: 3SAJ) into the cryo-EM envelopes, the angles between the global two-fold symmetry axis and the local two-fold axis of each NTD dimer reveal the large range of dimer motions (Fig. 2b,c); these can be grouped into three dominant modes of motion by principal component analysis (PCA) (Supplementary Video 1 and Methods).

## Domain un-swapping

Surprisingly, some classes, constituting a small proportion (approximately 20%) of particles, deviated from the established AMPAR organization by lacking the characteristic domain swap between the NTD and LBD tiers, in both active and resting states (Extended Data Fig. 5b,c). As a result, an A/B NTD dimer continued into an A/B LBD dimer (Fig. 2d and Supplementary Video 2), rather than swapping into an A/D LBD dimer. This atypical arrangement is observed in orphan GluD receptors^32^, but has not yet been described for any other ionotropic glutamate receptor (iGluR)^1^. As we demonstrate below, this symmetry switch is linked to the strength of the tetrameric B/D NTD interface and impacts gating transitions.

## NTD influence on GluA1 kinetics

**Extended Data Fig. 6:**
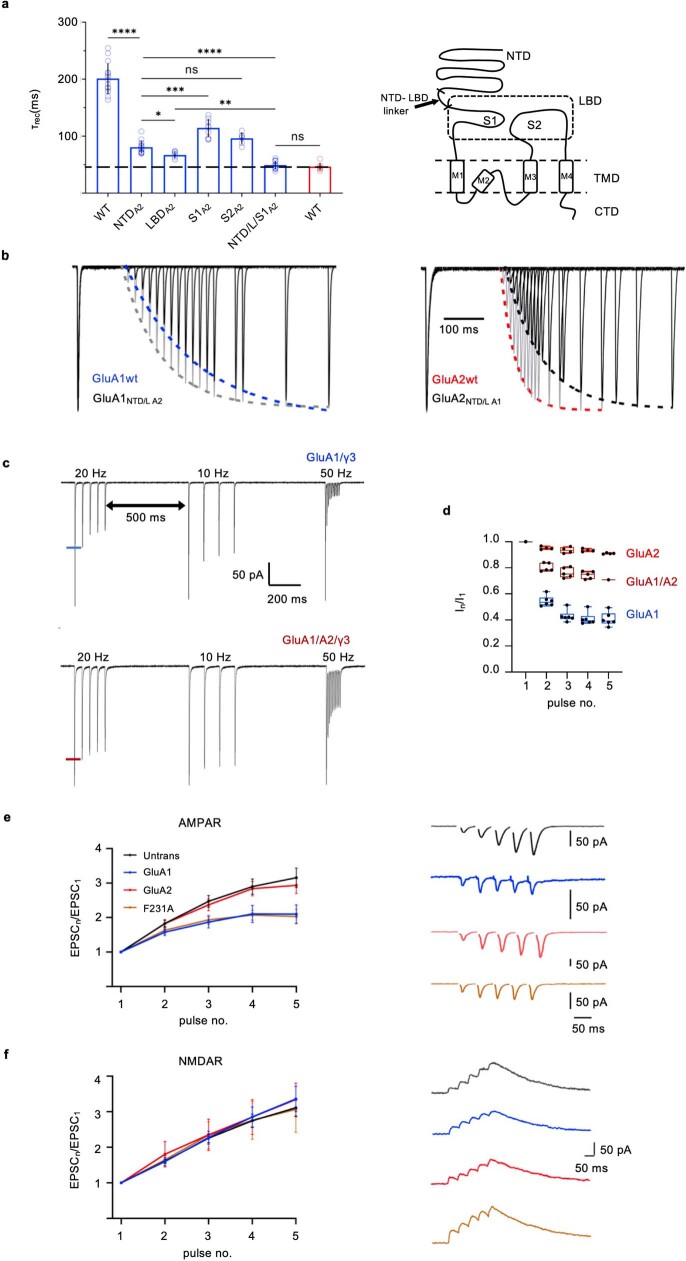
NTD flexibility tunes desensitization recovery and the AMPAR frequency response. **a**, Left: Desensitization recovery for various GluA1 (blue) and GluA2 (red) constructs (mean ± SD; Welch’s ANOVA (W_6,25.30_ = 102.3; p < 0.0001) with Dunnett’s multiple comparisons; ****p < 0.0001, ***p = 0.0005, **p = 0.0018, *p = 0.0347, see Extended Data Table 2). Right: Schematic showing NTD and LBD regions S1 and S2 exchanged between different constructs. **b**, Representative current responses showing recovery from desensitization from a single whole cell expressing GluA1 NTD_A2_ (left panel) and GluA2 NTD_A1_ (right). The ratios of the peak amplitudes (P_2_/P_1_) obtained in a paired-pulse protocol were plotted against the time and fitted with the single exponential function (black lines; τ = 109 ms and 101 ms for GluA1 NTD/L_A2_ and GluA2 NTD/L_A1_, respectively). For comparison, recovery profiles of GluA1 (blue; τ = 192 ms) and GluA2 (unedited-Arg; red; τ = 41 ms) are also provided, as exponential fits to representative single cell responses. **c**, Example outside-out recordings for GluA1 + γ3 (top) and GluA1/GluA2 + γ3 (bottom). Patches were stimulated with 10 mM glutamate delivered as 2 ms trains at three different frequencies (20, 10, and 50 Hz). **d**, Data summed up as box-and-whiskers plots; blue: GluA1 + γ3 (n = 6), red: GluA1/GluA2 + γ3 (n = 5); centre line, box, and whiskers represent median, 25th/75th centile and min/max values, respectively. **e**, Synaptic AMPAR recordings of paired pulse facilitation within a 20 Hz train in organotypic slice culture transfected with GluA1 (n = 14), GluA2 (n = 10), GluA2(F231A) (n = 16) or untransfected recorded simultaneously (n = 18). Peak current amplitudes of each EPSC were normalized to the first in the train (EPSC_n_/EPSC_1_). **f**, NMDAR-mediated PPF shows no difference between transfected and untransfected conditions. Data are presented as mean ± SEM. Example traces in panels e and f have timepoints containing the stimulation artefact removed. Source Data

A hallmark of GluA1 receptors is their uniquely slow recovery from non-conducting desensitized states, which will impact short-term synaptic plasticity at some synapses^9^. The kinetic difference from GluA2 is stark (GluA1 recovery time constant: 205 ± 34 ms (*n* = 17) versus GluA2: 48 ± 7 ms (*n* = 14)), and persists in the presence of TARPs (γ2, γ3) (Fig. 2e and Extended Data Table 2), which are known to speed GluA1 desensitization recovery^33,34^. Replacing NTD and LBD segments revealed that the NTD is a key contributor to this kinetic behaviour—GluA1 receptors harbouring the GluA2 NTD (GluA1 NTD_A2_ and GluA1 NTD/L_A2_) recover faster, whereas the GluA1 NTD substantially slowed recovery kinetics of GluA2 (Fig. 2e and Extended Data Fig. 6a,b). The impact of substituting the GluA2 NTD closely mirrored that of the GluA2 LBD, which directly gates the channel, whereas transferring the NTD together with LBD segment 1 (GluA1 NTD/L/S1_A2_) fully replicated GluA2 recovery kinetics (Extended Data Fig. 6a). The NTD specifically altered recovery, but not desensitization entry (Extended Data Table 2), which was completely reversed with LBD segment 2 (GluA1 S2_A2_). Together with the finding that NTD deletion accelerates desensitization recovery^35^, these results highlight NTD-LBD cooperation in AMPAR gating. These slow recovery kinetics cause greater depression of peak currents in response to a train of stimuli when comparing recombinant GluA1 with GluA2 or the GluA1/2 heteromer (Fig. 2f and Extended Data Fig. 6c,d). In addition, trains of electrical stimulation (20 Hz) at CA1 synapses show reduced paired-pulse facilitation (PPF) in pyramidal neurons transfected with GluA1 versus GluA2 (Extended Data Fig. 6e). Because NMDA receptor responses were unaffected in these conditions (Extended Data Fig. 6f), a postsynaptic contribution, involving the slower GluA1 kinetics, can be inferred.

**Extended Data Fig. 7:**
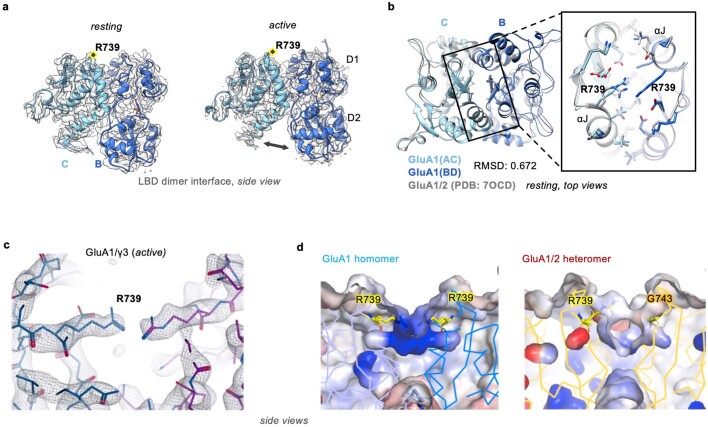
Features of resting and active GluA1/γ3 LBD dimers. **a**, Side view of cryo-EM density and model of GluA1/γ3 LBD dimer in resting and active states. **b**, Top of resting GluA1/γ3 LBD dimer interfaces aligned to those from a GluA1/2 heteromer (PDB: 7OCD), showing close similarity despite the presence of the R739 bridge. **c**, A zoomed view into the arginine bridge between R739 residues resulting from lack of an R/G editing site in active GluA1 supported by strong density shown for the open state as seen for R/G-unedited GluA2 LBD (PDB: 2UXA). A similar arrangement is also seen in the resting state (not shown). **d**, Electrostatic maps from APBS showing the dispersal of positive charge around R739 in GluA1 compared to the GluA1/2 heteromer, contoured from −10kT/e (red) to + 10kT/e (blue).

The NTD replacement hastened GluA1 recovery to a similar extent as two other established modulators: the aforementioned TARP^33,34^ and the Arg739Gly mutation in the GluA1 LBD dimer interface (Fig. 2e and Extended Data Fig. 7a)^36^. This arginine is naturally converted to glycine in GluA2–4 by RNA editing at the R/G site, speeding desensitization recovery. This switch is uniquely absent in GluA1 (ref. ^37^); the Arg739 side chains form an unusual bridge across the LBD dimer interface (Extended Data Fig. 7b,c), mirroring R/G-unedited GluA2 (ref. ^38^). The resulting positive electrostatic potential may cause repulsion between LBDs (Extended Data Fig. 7d), and thereby exert its role in slowing desensitization recovery. We provide further insights into this mechanism below. Together, our data reveal a functional role for the flexible GluA1 NTD tier.

## GluA2(F231A) triggers NTD dynamics

**Extended Data Fig. 8:**
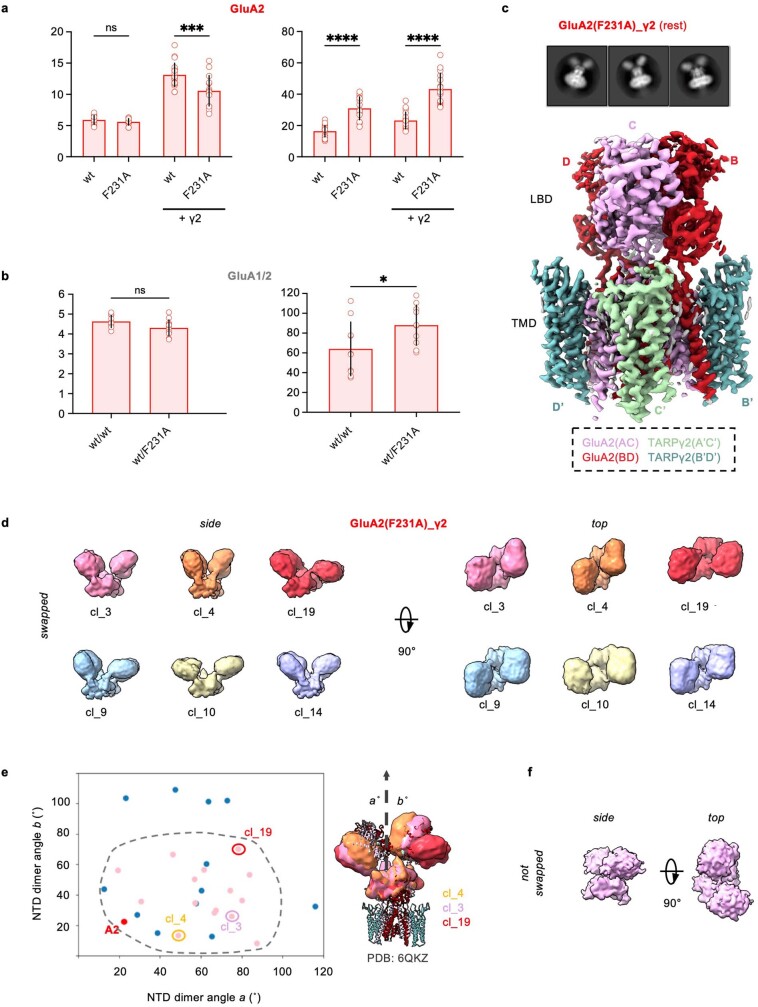
Disrupting the NTD inter-dimer interface. a, Summary graph for time constants of desensitization and recovery from desensitization in outside-out recordings of GluA2 (edited-Gly) with and without TARP γ2. Data are presented as mean ± SD. One-way ANOVA (F_3,54_ = 68.40; p < 0.0001)_des_; (F_3,65_ = 52.99; p < 0.0001)_rec_ with Sidak’s multiple comparison test (τ_des_: TARP-free: p = 0.8849; + γ2: ***p = 0.0001; τ_rec_: TARP-free: ****p < 0.0001; + γ2: ****p < 0.0001). Number of patches - τ_des_: 11 (wt), 12 (F231A), 20 (wt+ γ2), 15 (F231A + γ2); τ_rec_: 22 (wt), 13 (F231A), 18 (wt+ γ2), 16 (F231A + γ2). b, As A, for GluA1/GluA2 (unedited-Arg) heteromers (TARP-free). Data are presented as mean ± SD. Unpaired two-tailed Student’s t-test: *t*(20) = 2.055; p = 0.0531 (τ_des_), *t*(18) = 2.234; *p = 0.0384 (τ_rec_). Number of patches - τ_des_: 10 (wt/wt), 12 (wt/F231A); τ_rec_: 10 (wt/wt), 10 (wt/F231A). c, The resting state GluA2(F231A)/γ2 LBD-TMD map is shown together with representative 2D class averages, demonstrating the high resolution in the receptor core and the lack of NTD inter-dimer interface. d, Side (left) and top (right) views of representative NTD 3D classes from resting state GluA2(F231A)/γ2 show separation and splaying of the NTD dimers, but less splaying than GluA1/γ3. e, Conformational landscape of resting state GluA2(F231A)/γ2 (pink) and GluA1/γ3 (blue) in the space of splaying angles, with an A2-containing reference structure (PDB: 6QKZ; ‘A2’ red) shown for comparison, shows reduced splaying of GluA2(F231A)/γ2. Representative classes in d are circled with corresponding colors and shown overlaid on the reference structure (right). f, A GluA2(F231A)/γ2 3D NTD class from a non-swapped receptor. Source Data

In GluA2, the tetrameric NTD interface stabilizing the Y-shape^17^ is formed by a cluster of residues at the base of helix αG (Thr204-Val209), which is flanked by a cation–pi interaction between Phe231 (in αH) and Arg172 (in αF) on either side (Fig. 3a,b). The Phe231 to alanine mutation, which destabilizes this interface, slowed desensitization recovery of GluA2(F231A) (Fig. 3c), both in the absence and presence of TARPs (γ2 and γ3) (Extended Data Fig. 8a and Extended Data Table 2). Consistent with GluA2 location at the interface-forming B/D positions in AMPAR heteromers, recovery slowing is also seen in the heteromeric GluA1/GluA2(F231A) receptor (Extended Data Fig. 8b)^18,19^. NTD and LBD changes contributed additively: the R/G-unedited (Arg743) isoform harbouring a mutated B/D NTD interface (Phe231Ala) recovered the slowest (Fig. 3c). Moreover, at CA1 synapses, PPF of GluA2(F231A) more closely followed GluA1 than GluA2 wild type (Extended Data Fig. 6e).

To probe the relationship between NTD stability and gating further, we determined a cryo-EM structure of resting-state GluA2(F231A) fused to TARP γ2 (GluA2(F231A)/γ2; edited at the R/G site) (Fig. 3d and Extended Data Figs. 2c and 8c), a receptor combination permitting comparison with existing GluA2/γ2 structures^1^. Contrary to these, GluA2(F231A)/γ2 exhibited splayed NTD dimers (Fig. 3d,e), reminiscent of GluA1, although the conformational space of GluA2(F231A)/γ2 appeared narrower (Extended Data Fig. 8d,e). Nevertheless, the tetrameric B/D interface was lost throughout 3D classes, highlighting a central role of the GluA2-specific Phe231 in establishing the canonical AMPAR Y-shape, and the impact of this arrangement on desensitization recovery. As in GluA1, we obtain particles (about 10%) without domain swap between NTD and LBD (Extended Data Fig. 8f), drawing a further link between NTD B/D interface stability and domain swapping. As the NTDs form tight dimers in the nanomolar range^39,40^, the domain swap (and its reversal) probably involves the LBD dimers, which are substantially more flexible^41,42^.

## NTD-driven desensitization mechanism

**Fig. 4:**
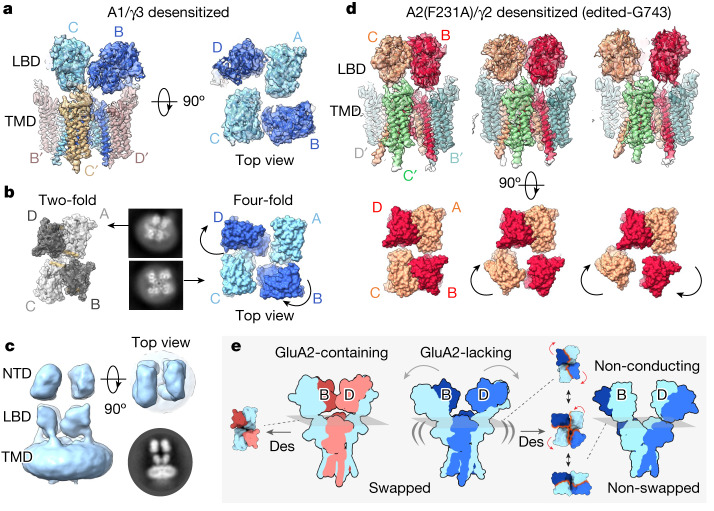
Desensitization behaviour of GluA1/γ3 and GluA2(F231A)/γ2. **a**, 3D map and model of desensitized GluA1/γ3 LBD–TMD (cf-3 in Extended Data Fig. 9d,e) approaches a four-fold symmetric LBD layer (side view, left; top view, right). **b**, Both 2D class averages (middle) and fitted structures (flanking) highlight divergence from two-fold symmetry of A2-containing receptors (PDB: 7QHH) to a pseudo four-fold symmetry in GluA1 (cf-4). Main movements are of B/D subunits (Methods and Extended Data Fig. 9e). **c**, NTDs belonging to four-fold GluA1/γ3 conformations exhibit parallel configurations as also apparent in 2D classes (bottom right). **d**, The main conformations (cf1–3) of desensitized R/G-edited (Gly743) GluA2(F231A)/γ2 show a similar behaviour to GluA1, together with greater movement of A/C subunits (top, side view of maps and models; bottom, top view of models). Curved arrows indicate LBD subunit motions. **e**, Overview of main findings: stabilized by the tetrameric NTD interface, GluA2 receptors (left panel) undergo relatively subtle conformational changes in the LBD tier upon desensitization, remaining dimeric (top view, left). In GluA1 (‘A2-lacking’; middle and right), rupture of the B/D NTD interface (arrows) destabilizes B/D LBDs, rendering them more mobile (indicated by double brackets). This mobility is enhanced in desensitized states, triggering LBD switching to four-fold symmetry and dimer swapping (top views), which give rise to non-swapped, non-conducting AMPARs. These reconfigurations extend into the NTD, impacting synaptic receptor anchoring (Extended Data Fig. 10g). Red arrows denote LBD motions, whereas wavy orange lines indicate LBD dimer interface regions. Des, desensitization.

**Extended Data Fig. 9:**
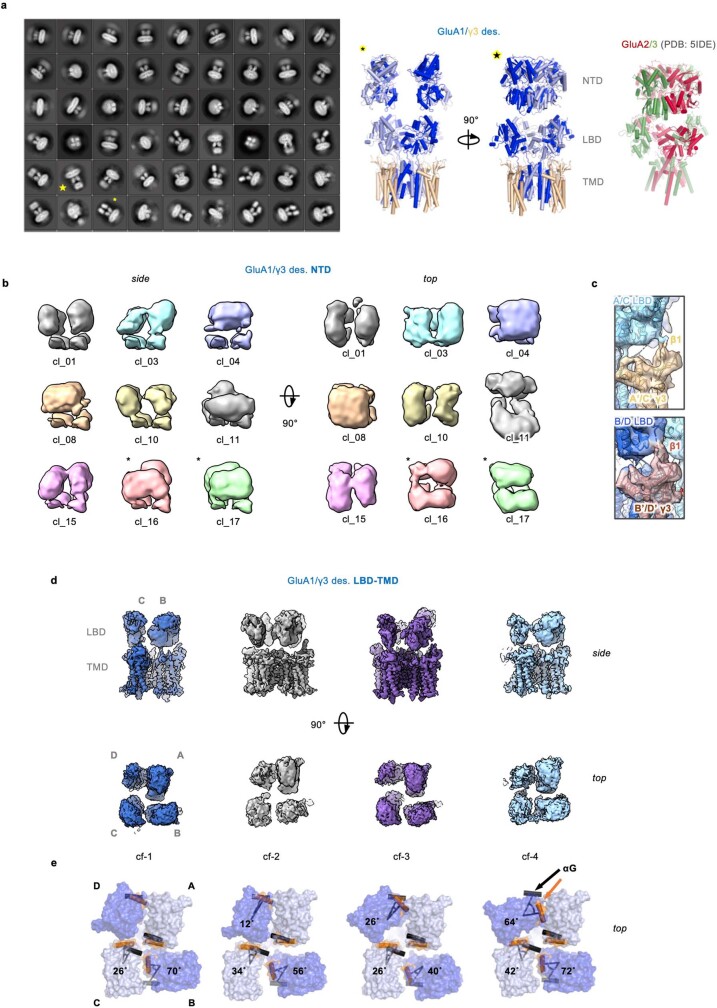
Variability within GluA1/γ3 desensitized states. **a**, Left: 2D classes from desensitized GluA1/γ3 show various states, some of which have well-defined, parallel NTD layers and/or separated, 4-fold symmetric LBDs. Middle: Model of desensitized GluA1/γ3, depicting the stark changes throughout the extracellular region (fitting EM map shown in Fig. 4c). Two views with similar appearance to the 2D class averages are highlighted by asterisk and star, respectively, in both panels. Right: For comparison, the GluA2/GluA3 heteromer structure^45^ (PDB: 5IDE; GluA2: red, GluA3: green), adopting a similar ‘O-shape’ organization of the NTD tier obtained by cysteine cross-linking of the NTDs in an apo receptor, is provided. **b**, Side (left) and top (right) views of representative NTD classes from one of the desensitized GluA1/γ3 LBD-TMD classes show a range of parallel NTD configurations that appear to be in both swapped and non-swapped (asterisked) arrangements. **c**, Zoom into TARP γ3 contacts with the LBD in GluA1/γ3 3D class_2 (cl_2). Contacts between the β1 loop and the LBD lower lobe is seen for both TARPs (A’/C’) and (B’/D’) under desensitizing conditions, but the precise interaction site on the LBD D2 lobe is unresolved. **d**, Four representative conformations (cf1-4) from 3D variability analysis are shown, illustrating extensive dimer rupture and divergence from 2-fold symmetry, approaching 4-fold symmetry in some cases. **e**, Rotations of the LBDs are quantified for these four desensitized GluA1/γ3 LBD-TMD conformations (same order as in panel d), showing varying degrees of rotation of B/D subunits relative to their positions in a canonical 2-fold symmetric A2-containing desensitized structure (PDB: 7QHH; reference) around an axis through the start of the M1 linker (**Methods**). Helix G used for measurement is highlighted in orange for GluA1/γ3 and in grey for the reference. Cf-4 is closest to 4-fold symmetry, with the αG angles for chains B and D approaching 75°.

**Extended Data Fig. 10:**
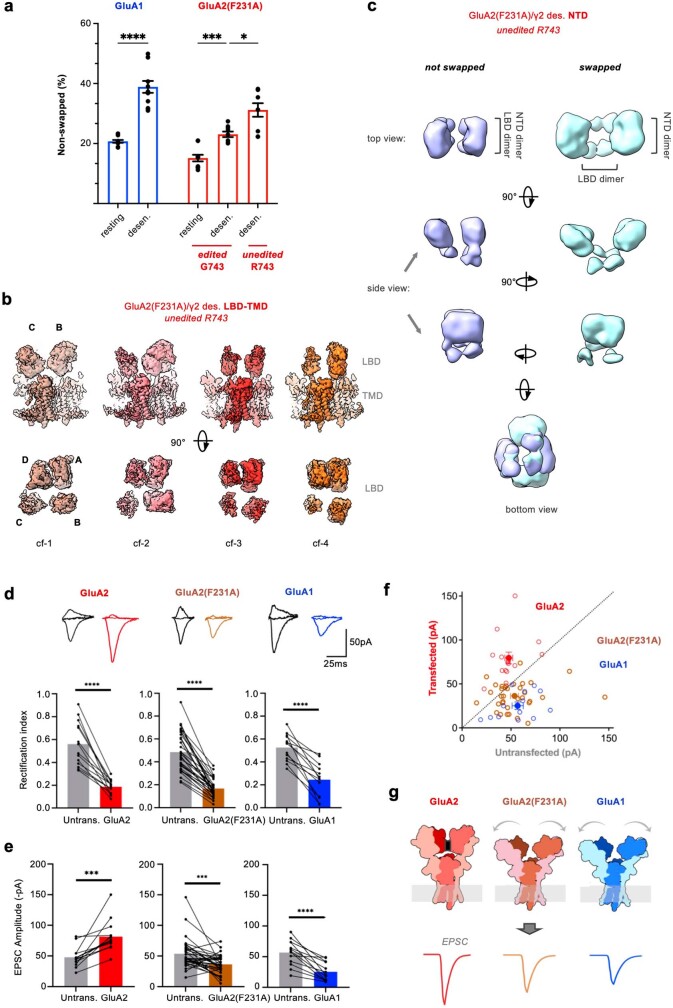
Further variability within GluA1/γ3 and GluA2(F231A)/γ2 desensitized states and its impact on synaptic physiology. **a**, Quantification of the percentage of non-swapped dimers across different datasets is shown based on a consensus classification approach (see Methods). Black points are from individual classifications, bars represent mean values and error bars represent standard error of the mean for resting GluA1/γ3 (20.3 ± 0.5%, n = 12), desensitized GluA1/γ3 (38.9 ± 2.0%, n = 12), resting R/G-edited GluA2(F231A)/γ2 (Gly743; 15.2 ± 1.1%, n = 10), desensitized R/G-edited GluA2(F231A)/γ2 (Gly743; 23.1 ± 0.9%, n = 9), and desensitized R/G-unedited GluA2(F231A)/γ2 (Arg743; 31.2 ± 2.3%, n = 9). Welch’s one-way ANOVA (W_4,20.73_ = 30.46; p < 0.0001) with Dunnet’s T3 multiple comparison test (****p < 0.0001, ***p = 0.0001, *p = 0.0191). **b**, Four main conformations (cf1-4) of desensitized R/G-unedited GluA2(F231A)/γ2 (R743) (top: side views; bottom: top view onto LBD tier). Compared to the edited (Gly743) GluA2 F231A isoform, greater splitting of the D/A dimers are evident together with larger motions of the C/B subunits, which is pronounced in cf-2 and cf-3. **c**, Two representative NTD classes from the unedited (R743) GluA2(F231A)/γ2 desensitized state with the clearest LBD arrangement, showing non-swapped and swapped arrangements. **d**, Synaptic expression of transfected AMPARs in organotypic slice shown by a reduction in Rectification Index relative to a neighboring untransfected neuron recorded simultaneously: GluA2Q (paired two-tailed t-test: t = 8.14, n = 15, p < 0.0001), GluA2Q(F231A) (t = 12.6, n = 34, p < 0.0001) or GluA1 (t = 7.13, n = 13, p < 0.0001). Representative traces of EPSCs at −60, 0 and +40 mV holding potential shown above corresponding bar graphs. **e,f**, EPSC peak amplitude increases in GluA2Q expressing neurons (untransfected: 48 ± 15 pA, GluA2: 79 ± 26 pA; paired two-tailed t-test t = 4.82, n = 15, p = 0.0003), and decreases in GluA2Q F231A (untransfected: 53 ± 24 pA, GluA2(F231A): 36 ± 15 pA; t = 3.75, n = 34, p = 0.0007) and GluA1 (untransfected: 57 ± 21 pA, GluA1: 25 ± 15 pA; t = 5.72, n = 13, p < 0.0001) expressing neurons relative to their neighboring untransfected cell. **g**, Proposed model of how the enhanced mobility of the NTD in GluA2(F231A) and GluA1 may impact synaptic receptor anchoring, and thereby reduce the EPSC observed upon presynaptic release. Black rectangle in wildtype GluA2 marks the tetrameric interface, absent or disrupted in the other two receptors. Source Data

AMPAR desensitization is linked to the stability of the LBD D1 dimer interface^42^. A rearranged D1 interface, which uncouples LBD tension from the gate, is the major conformational change in desensitized GluA2 complexes^21,24,43^. The overall Y-shape and two-fold LBD organization remain largely intact, when stabilized by associated auxiliary subunits^21,24,43,44^. Desensitized GluA1/γ3 substantially deviates from this behaviour and, despite the limited NTD and LBD resolution of this highly dynamic state, we could trace major transitions throughout the receptor (Fig. 4a–c). First, the GluA1 LBDs splay into pseudo four-fold symmetry in some 3D classes (Fig. 4a,b), reminiscent of desensitized kainate receptors, and of GluA2 receptors lacking auxiliary subunits^29,30^. Second, the NTD dimers translate into a parallel configuration, where some classes adopt an ‘O-shape’ conformation, as described for heteromeric GluA2/3 and GluA2/4 NTDs (Fig. 4c, Extended Data Fig. 9a,b and Supplementary Video 3)^45^. Interestingly, these transitions take place despite the presence of TARPs, whose extracellular loops remain associated with the LBDs, and accompany their motions (Fig. 4a and Extended Data Fig. 9c). Last, the fraction of non-domain-swapped receptors is visibly increased (Extended Data Fig. 10a), compared with the resting state.

As apparent in 3D classes, transition of the LBDs towards four-fold symmetry largely involves rotation of the B/D subunits (Fig. 4b, Extended Data Fig. 9d,e and Supplementary Video 4). The B/D LBDs undergo up to 90° rotation along an axis perpendicular to the M1 gating linkers, with the M3 and M4 linkers accommodating this motion. The appearance of non-domain-swapped receptors reaches approximately 30% and is greatest in the class closest to four-fold symmetry. Together with a study implicating subtler GluA2 LBD rearrangements in desensitization^46^, a complete rupture of the LBD dimer interface may lead to deeply desensitized states.

The role of B/D NTD interface stability in this transformation is supported by a desensitized (R/G-edited) GluA2(F231A) structure (Fig. 4d). The NTD dimers of the mutant receptor also transit into parallel O-shape conformations, and the LBDs depart from two-fold symmetry, which again is coupled to an increase of non-domain-swapped receptors (relative to the resting-state receptor; Extended Data Fig. 10a). Rotations are larger for one of the two LBD dimers (C/B versus A/D), and movement of the B/D subunits is less pronounced than in GluA1 (Fig. 4d), perhaps due to subunit-selective contacts between the NTD and LBD tiers^19^.

Together, this suggests that lack of the tetrameric NTD interface increases mobility of the LBDs, facilitating their transition towards four-fold symmetry. This symmetry switch enables the re-alignments of the LBD dimers that lead to non-swapped receptors, and, in turn, slowed desensitization recovery (Fig. 4e). Transition into non-swapped LBD dimers probably involves relaxation of the critical M3 gating linkers of the B/D subunits, as observed in an NTD-deleted NMDAR structure^47^, thereby entering non-conducting (‘deeply desensitized’) conformations (Figs. 2e and 4e).

## Role of the R/G editing site in recovery

A desensitized GluA2(F231A) structure unedited at the R/G site (Arg743) exhibited more separation of the LBD dimers and an approximately 10% increase of non-swapped receptors relative to the edited Gly743 isoform, approaching GluA1 (Extended Data Fig. 10a–c). This suggests that the re-assembly of desensitized A/D LBDs is attenuated by Arg743 through charge repulsion (Extended Data Fig. 7d), resulting in a greater proportion of A/B LBD dimers, and thus non-swapped receptors. Closer analysis of the three sets of desensitized structures (Methods) reveals a gradual transition of the LBD tier into four-fold symmetry, which is greatest for GluA1 and smallest for the edited GluA2 mutant: GluA1 > GluA2(F231A)-Arg743 > GluA2(F231A)-Gly743. Hence, communication between the NTD tier and electrostatics at the R/G site in the LBD orchestrates AMPAR desensitization recovery.

## NTD dynamics impact synaptic anchoring

As the NTDs play a role in AMPAR anchoring at synapses, GluA1 NTD dynamics might underlie its unique, activity-dependent synaptic delivery^4,5,11,48,49^. In the absence of suitable mutations stabilizing the GluA1 NTDs, we assayed the effect of the GluA2-destabilizing F231A mutation on transmission at hippocampal CA1 synapses. Both GluA2 wild type and NTD mutant translocated into synapses, as evidenced by a change in their rectification indices (Extended Data Fig. 10d)^4,5,11^. Consistent with earlier data^5^, transfection of wild-type GluA2 increased excitatory postsynaptic currents (EPSCs), relative to a nearby untransfected neuron, which was not seen with GluA1 (Extended Data Fig. 10e). By contrast, EPSCs from neurons expressing GluA2(F231A) were reduced, closely mirroring the behaviour of GluA1 (Extended Data Fig. 10e,f)^5,11^. Therefore, NTD conformational flexibility is a key contributor to the stable insertion of AMPARs at synapses (Extended Data Fig. 10g), and may underlie the activity-dependent recruitment of GluA1 during LTP.

## Conclusion

In summary, we demonstrate that GluA1 departs from the canonical A2-containing AMPAR organization and gating. NTD sequence divergence leads to loss of the tetrameric B/D interface control hub, enabling subunit re-alignments associated with gating regulation (Fig. 4e), which may also arise in other non-GluA2 AMPARs. This impacts the AMPAR frequency response and could thereby dampen otherwise excessive Ca^2+^ influx, which is highly excitotoxic if unchecked^2^. Moreover, in addition to shaping kinetics, NTD conformational flexibility plays a role in short-term plasticity, which involves a mechanism associated with desensitized conformations^50^, and probably contributes to the unique recruitment and anchoring of GluA1 following LTP^5,11^. In addition to their proposed clustering function^51–53^, synaptic NTD interaction partners may impact NTD conformations and tune AMPAR response kinetics. With the diverse AMPAR subunits still to be investigated, we expect that other non-GluA2 NTDs will hold further surprises in their regulation of AMPAR subunit organization, gating and synaptic transmission.

## Methods

### Complementary DNA constructs

All cDNA constructs were produced using IVA cloning^54^. To achieve preferred stoichiometry of TARP, tandem constructs were used for all structural studies in this work, unless stated otherwise: γ3 was fused to GluA1 and γ2 was fused to GluA2. GluA1_γ3 tandem was cloned by fusing TARP γ3 (rat cDNA sequence) to the C terminus of GluA1 (rat cDNA sequence, flip isoform) in pRK5 vector with a Gly-Ser-Gly-Ser-Gly linker sequence. GluA1 has a FLAG tag at its N terminus right after the signal peptide, and an EGFP tag together with a 3C protease cleavage site were also added to the C terminus of γ3 for visualization and purification.

GluA2_γ2 tandem was cloned in a similar way: TARP γ2 (rat cDNA sequence) was cloned to the C terminus of GluA2 (rat cDNA sequence, flip isoform, R/G-edited, Q/R-edited) with a Gly-Ser-Gly-Ser-Gly linker in pRK5 vector. GluA2 was FLAG tagged and γ2 was EGFP tagged, and a 3C protease cleavage site was added between γ2 and EGFP to be able to remove the tag during purification. F231A mutants used in this work were cloned using IVA cloning based on wild-type constructs, and both R/G-edited and unedited constructs were used for F231A desensitized samples.

### Electrophysiology constructs

Chimeric proteins were made by substituting parts of the rat GluA1 receptor with the corresponding parts of the rat GluA2. Receptors are flip variants, and GluA2 is unedited at both 586 Q/R and 743 R/G, unless stated differently. The new chimeras were cloned in pIRES-mCherry construct. Two constructs were made for NTD substitution, one including the peptide linker between NTD and LBD and one without the linker. GluA1 NTD/L_A2_ was made by replacing the residues 1–390, whereas GluA1 NTD_A2_ construct was made by replacing residues 1–373, and these were replaced with the ones from GluA2. LBD domain substitution (GluA1 LBD_A2_) was done by replacing residues 391–778 from GluA1 with the corresponding sequence of GluA2 (the short M1–M2 intracellular loop was thus also replaced in this construct, the rest of the TMD part was conserved). GluA1 S1_A2_ and GluA1 S2_A2_ constructs were made by replacing GluA1 residues 391–472 and 636–778, respectively. GluA1 NTD/L/S1_A2_ construct was made by replacing residues 1–472 from GluA1 with the corresponding residues of GluA2.

### Expression and purification of GluA1/γ3 and GluA2(F231A)/γ2

For all systems studied in this work, the corresponding plasmid was transfected into Expi293 cells. To prevent AMPA-mediated excitotoxicity, AMPAR antagonists ZK200775 (2 nM, Tocris, catalogue no. 2345) and kynurenic acid (0.1 mM, Sigma, catalogue no. K335-5G) were added to the culture medium. At 36–44 h posttransfection, cells were collected and lysed for 3 h in lysis buffer containing 25 mM Tris pH 8, 150 mM NaCl, 0.6% digitonin (w/v) (Sigma, catalogue no. 300410-5G), 1 mM PMSF and 1 × Protease Inhibitor (Roche, catalogue no. 05056489001). Insoluble material was then removed by ultracentrifugation (41,000 r.p.m., 1 h, rotor 45-50 Ti) and the clarified lysate was incubated with anti-GFP beads for 3 h. After washing with glyco-diosgenin (GDN; Anatrace, catalogue no. GDN101) buffer (25 mM Tris pH 8, 150 mM NaCl, 0.01% GDN), the protein was eluted from the beads by digestion with 1 mg ml^−1^ 3C protease at 4 °C overnight. Eluted fractions were incubated with FLAG beads (Sigma, catalogue no. A2220) for 1.5 h and washed three times with washing buffer (25 mM Tris pH 8, 150 mM NaCl, 0.01% GDN, 1 mM MgCl_2_, 1 mM ATP). Finally, the complex was eluted using 0.15 mg ml^−1^ 3 × FLAG peptide (Millipore, catalogue no. F4799) in GDN buffer (25 mM Tris pH 8, 150 mM NaCl, 0.01% GDN). For protein used for active-state sample preparation, an extra 10 mM CaCl_2_ was added to the elution buffer. Eluted fractions were pooled and concentrated to 2.6–3 mg ml^−1^ for cryo-EM grid preparation.

### Cryo-EM grid preparation and data collection

Cryo-EM grids were prepared using an FEI Vitrobot Mark IV. For the resting state, protein was incubated with 300 μM ZK200775 (Tocris, catalogue no. 2345) for at least 30 min on ice before freezing. For the active state, GluA1/γ3 homomeric complex, protein was first incubated with 300 μM cyclothiazide (Tocris, catalogue no. 0713) for at least 30 min on ice and then quickly mixed with 1 M l-glutamate stock solution to a final concentration of 100 mM before loading onto the grids. For desensitized structures, 10 mM quisqualate (Tocris, catalogue no. 0188) was quickly added to protein to a final concentration of 1 mM before loading. Quantifoil Au 1.2/1.3 (300 mesh) or Quantifoil Cu 1.2/1.3 (300 mesh) grids were glow-discharged for 30 s before use. A 3 μl sample was applied to the grids, blotted for 4.5–6 s at 4 °C with 100% humidity and plunge-frozen in liquid ethane.

All cryo-EM data were collected on an FEI Titan Krios operated at 300 kV, equipped with a K3 detector (Gatan) and a GIF Quantum energy filter (slit width 20 eV). Videos at 1.5–2.5 μm underfocus were taken in counting mode with a pixel size of 1.06 Å per pixel or 0.826 Å per pixel. A combined total dose of 50 e/Å^2^ was applied with each exposure and 50 frames were recorded for each video. All datasets were collected using EPU2.

### Cryo-EM data processing and model building

Dose-fractionated image stacks were first motion-corrected using MotionCor2 (ref. ^55^) in RELION4.0 (ref. ^56^). Corrected sums were then imported into CryoSPARC^57^ and used for contrast transfer function (CTF) estimation by Patch CTF estimation. Blob Picker was used to pick particles from the first 400 micrographs. Picked particles were extracted (down-sampled by a factor of 4) and 2D classified to get good class averages for Template Picker. After template-based particle picking, all particles were extracted with a binning factor of 4 and 3D classified to remove bad particles by using Heterogeneous Refinements with initial models coming from two different Ab-initio Reconstruction jobs. The first job was used to generate a proper model for AMPAR; particles from the first 400 micrographs were imported to generate several initial models and the best one was selected as the good particle template. Then, the same Ab-initio Reconstruction job was cloned and stopped manually after the first iteration to generate models for noise; all five models were used as bad particle templates afterwards. Four to five rounds of Heterogeneous Refinement on all extracted particles were then performed by using the one good model and five noise models as references.

Good particles were finally selected and scaled back to a binning factor of 2 for Homogeneous Refinement. Dynamic masks were used all the way during Heterogeneous and Homogenous Refinements. Next, particle coordinate files generated from Homogeneous Refinement were converted to RELION star files by using the Python script csparc2star.py (ref. ^57^) with the flag --swapxy. Particles were re-extracted with a binning factor of 2 in RELION and refined using the masks generated from Homogeneous Refinement in CryoSPARC.

Additional 3D classifications focused on the LBD–TMD region were performed to separate different conformations or further clean up the datasets. Selected particles were refined and postprocessed for the following Bayesian polishing and CTF refinement (only for high-resolution maps). During polishing, particles were re-scaled to original pixel size if the map resolution from the last refinement reached Nyquist. Final reconstruction was performed after polishing and CTF refinement; focused refinement on the LBD–TMD region or TMD region alone was carried on afterwards. C1 symmetry was applied through all the processing until here.

Focused classifications on TARP loops were performed from particles used for final reconstruction. First, a 3D refinement with C2 symmetry was done focusing on the LBD–TMD region, then symmetry expansion was applied on the aligned particles. The newly generated star file was used as an input for 3D classifications without alignment, focusing on the extracellular loop regions of TARPs at the A/C or B/D side; different class numbers and regularization parameter T were screened to have the best separation of different conformations. Particles from individual 3D classes were then refined separately.

3D classifications on the NTD region were also performed using particles from the final reconstructions. Particles were first down-sampled to 2–3 Å per pixel and refined. Then, a soft mask at the NTD region was used to subtract the signal of the LBD–TMD region from the particles. Subtracted particles were clipped and re-centred afterwards. 3D classifications without alignment were performed on the subtracted particles, no masks were applied for these classifications and default regularization parameter T and up to 30 classes were given.

Model building and refinement for high-resolution systems were performed using Coot^58^, PHENIX^59^ real-space refinement and Refmac-Servalcat^60^. C1 maps of LBD–TMD were used for general building of all models. Corresponding domains from published structures (PDB: 6QKC, 7OCF and 6DLZ) were used as initial models and first rigid-body fitted into the map using UCSF chimera (http://www.rbvi.ucsf.edu/chimera) and then refined by PHENIX real-space refinement. Afterwards, manual refinement was performed in Coot to further refine the geometry. Several rounds of PHENIX real-space refinement and manual refinement were performed iteratively. Finally, the models were refined against unsharpened and unweighted half maps using the Refmac-Servalcat pipeline. The reference structure restraints were prepared with ProSmart^61^ using AlphaFold2 predicted models from the Alpha Fold DB^62,63^. Water molecules were detected using Fo-Fc maps. Model validation was performed with MolProbity^64^. All graphics figures in the paper were prepared using UCSF Chimera, UCSF ChimeraX (https://www.rbvi.ucsf.edu/chimerax) or PyMOL (http://www.pymol.org). Pore radius was calculated using a plugin version of HOLE^65^ in Coot.

Low-resolution models of desensitized structures were created by rigid-body fitting of domains in Chimera and jiggle fitting in Coot^66^, followed by all-atom refinement in Coot with Geman–McClure self-restraints (option 4.3)^66^. For the GluA1/γ3 desensitized models, we used the LBDs from our open-state model and the TMD from our resting-state GluA1/γ3 LBD–TMD model. For GluA2(F231A)/γ2, we used the TMDs from our resting-state model and LBDs from a published quisqualate-bound GluA2 flop, R/G-edited LBD structure (PDB: 1MM7)^67^, with the flip cassette and upstream arginine (unedited R/G) from the corresponding published GluA2 LBD structure (PDB: 2UXA)^38^ or the flip cassette and upstream glycine (edited R/G) from our resting-state GluA2(F231A)/γ2 LBD–TMD model. DeepEMhancer^68^ was used to help with interpretability of low-resolution maps, including for Fig. 4, but not directly in model building or refinement.

The proportions of swapped versus non-swapped receptors were quantified by a classification approach based on the idea of consensus^69^. As it is known that discrete classification is inherently unstable and the results can vary across similar runs and also with the numbers of classes, RELION classifications were repeated in triplicate with different numbers of classes (from 3 to 30) and stability of percentages was assessed by comparing across the different runs as well as analysing co-migrating particle subsets between pairs of classifications with the same number of classes using a new protocol implemented in Scipion and Xmipp^70^. Final means and standard errors come from the most consistent sets of runs (containing between 6 and 20 classes), taking *n* as the total number of consistent runs.

### 3D variability analysis of desensitized conformations

Approximately 1.14 million refined GluA1/γ3 desensitized particles at 1.325 Å per pixel were imported into CryoSPARC^57^ from RELION to perform 3D variability analysis (3DVA)^71^. Multiple rounds of heterogeneous refinement were then performed to remove poorly aligning particles and produced around 745,100 particles. Non-uniform refinement^72^ of these particles using a mask covering the LBD and TMD domains yielded a reconstruction to around 2.7 Å resolution. Subsequent 3DVA (using the default settings except for six modes and filter resolution to 3.5 Å) was performed using the refined particles with a mask covering the entire molecule. Using the 3DVA display job in CryoSPARC, each mode was clustered into five clusters and local refinement was performed on each set of extreme clusters. Models were rigid-body fitted into each refined cluster and refined in PHENIX for the higher-resolution TMD and Coot with Geman–McClure self-restraints for the full LBD–TMD system. A similar procedure was applied to the two desensitized GluA2(F231A)/γ2 datasets using particles polished to 1.4455 Å per pixel, except that PHENIX real-space refinement was not applied as the TMDs did not reach as high resolution.

### Analysis of conformational changes in fitted resting NTD models

NTD dimers from published crystal structures of GluA1 and GluA2 NTDs (PDB: 3SAJ and 3HSY) were fitted to NTD class averages from resting-state GluA1/γ3 and GluA2(F231A)/γ2 using Chimera and chains were relabelled to match in PyMOL. For analysis of tilt angles and video creation, an A2-containing reference structure (PDB: 6QKZ) was aligned to a member of each set.

Tilt angles were analysed in the ProDy Python API (v.2.3.1 under development and available on GitHub)^73^. The angles between the second principal axis of each NTD dimer that ran along the dimer two-fold axis and the first principal axis of the reference structure were calculated as the arccosine of the dot product of normal principal axis vectors, with some angles subtracted from 180° if the NTD dimer was pointing down towards the LBD. Angles for resting GluA1/γ3 and GluA2(F231A)/γ2 were analysed separately in their own reference frames and plotted together afterwards. Non-swapped classes were excluded for simplicity.

Principal movements within these NTD layers were analysed using PCA in ProDy v.2.3.1 using a Calpha atom ensemble from all fitted models without alignment, including the rotated NTDs corresponding to non-swapped receptors. The first three eigenvalues of the positional covariance matrix were taken as the principal components (PCs) 1–3. PC1 revealed a rotation of the whole NTD layer relative to the rest of the receptor, given the classes come from classification without alignment, capturing the transition from swapped to non-swapped receptors. PC2 revealed a tilting of the whole NTD layer towards and away from the LBD layer. PC3 captured a varying concerted separation and tilting of NTD dimers related to splaying. The analysis was also repeated without the non-swapped classes and with alignment, revealing high-correlation cosine overlaps for all components besides PC1.

Supplementary Video 1 was created by adding each of the PC vectors individually to the average structure with a range of scaling factors in each direction related to the variation in the data, creating a set of 20 new structures to illustrate each of the motions, which were repeated to start and end with the average conformation. PC1 was scaled to a maximum root mean squared deviation (r.m.s.d.) of 20.0 Å in each direction, in line with projection of the ensemble onto PC1. PC2 and PC3 were scaled by the same scale factor of 0.076, giving r.m.s.d. values of 9.85 Å and 6.78 Å.

### Analysis of desensitized LBD rotations

The LBD rotations of all six desensitized models were analysed together in PyMOL. These models and an A2-containing reference desensitized structure (PDB: 7QHH) were first aligned to an A2-containing structure with its global symmetry axis aligned to the *z* axis (PDB: 4UQJ).

The centres of mass (COMs) of residues that connect the LBDs to the LBD–TMD linkers were visualized with pseudoatoms and K501 in GluA1 and K505 in GluA2 at the beginning of the S1–M1 linker were found to be stable in all chains, and these linkers often had clear vertical density, suggesting they act as axes of rotation. Accordingly, the COMs of all residues K501 and K505 in each chain were used as reference points for defining an axis parallel to the *z* axis.

The helix G COMs for each chain of each structure individually were calculated as the proxy points for the rotation. The combined COMs of helix G from each chain across all structures were also calculated to give reference points for finding the average position in *z* along the rotation axes for calculating rotation angles. The reference point for each subunit was given by the *x* and *y* values of the combined K501/K505 COM and the *z* value of the combined helix G COM. The angle was then calculated using the individual helix G COM of a given structure, the reference point and the helix G COM of the corresponding chain in the reference desensitized structure.

### Electrophysiology

HEK293T cells (ATCC: catalogue no. CRL-11268, RRID: CVCL_1926, Lot 58483269: identity authenticated by short tandem repeat analysis, mycoplasma negative), cultured at 37 °C and 5% CO_2_ in DMEM (Gibco; high glucose, GlutaMAX, pyruvate, catalogue no. 10569010) supplemented with 10% FBS (Gibco) and penicillin/streptomycin, were transfected using Effectene (Qiagen) according to the manufacturer protocol. Then, 30 μM 2,3-dioxo-6-nitro-1,2,3,4-tetrahydrobenzo[f]quinoxaline-7-sulfonamide (NBQX; Tocris, catalogue no. 1044; or HelloBio, catalogue no. HB0443) was added to media posttransfection to avoid AMPAR-mediated toxicity. The transfection ratio of AMPAR/TARP was 1:4. For heteromeric recordings, 20 μM IEM 1925 dihydrobromide (Tocris, catalogue no. 4198) was added to the extracellular solution to limit the contribution of GluA1 homomers.

Recording pipettes were pulled with a P-1000 horizontal puller (Sutter Instruments) using borosilicate glass electrodes (1.5 mm outside diameter, 0.86 mm inside diameter, Science Products). Electrode tips were heat-polished with an MF-830 microforge (Narishige) to final resistances of 2–4 MΩ (whole cell) and 6–12 MΩ (outside-out patches). Electrodes were filled with internal solution containing (in mM): CsF (120), CsCl (10), EGTA (10), HEPES (10), Na_2_-ATP (2) and spermine (0.1), adjusted to pH 7.3 with CsOH. The extracellular solution contained (in mM): NaCl (145), KCl (3), CaCl_2_ (2), MgCl_2_ (1), glucose (10) and HEPES (10), adjusted to pH 7.4 using NaOH.

Currents were recorded with an Axopatch 700B amplifier (Molecular Devices). Recordings were prefiltered at 10 kHz with a 4-pole Bessel filter (amplifier built-in), sampled at 100 kHz with the Digidata 1322A (Molecular Devices), stored on a computer hard drive and analysed using pClamp 10 software pack (Molecular Devices).

On the day of recording, cells were plated on poly-l-lysine-treated glass coverslips. Fast perfusion experiments were performed with a two-barrel theta tube glass cut to a diameter of approximately 300 µm. The theta tube was mounted on a piezoelectric translator (Physik Instrumente) and command voltage (9 V) was filtered with a 250 Hz Bessel filter to reduce mechanical oscillations. The theta tube was filled with pressure-driven solutions (ALA Scientific Instruments). Applied pressure on solutions for the lifted cell protocol was kept low (around 2,000 Pa) for patch stability. Speed of solution exchange at the theta tube interface was measured as 20–80% rise time of the current generated with 50% diluted extracellular solution and was on average about 300 µs. Cells were voltage-clamped at −60 mV (voltage not corrected for junction potential of 8.5 mV). Series resistance in a whole-cell recording was never higher than 8 MΩ and was compensated by 80–90%.

Recovery from desensitization was measured with a two-pulse protocol. Conditioning pulse of 10 mM glutamate with duration of 100 ms (GluA1 constructs, GluA1/GluA2 heteromers) or 200 ms (GluA2 constructs) was followed by 10 ms glutamate pulses delivered at intervals increasing by 20 ms (GluA1) or 10 ms (GluA2, GluA1/GluA2). Desensitization time constants were obtained by fitting current decay (Chebyshev algorithm, built in Clampfit 10.2, Molecular Devices) of the glutamate application (100 or 200 ms) from 90% of the peak to the baseline/steady-state current with one (TARP-free GluA1) or two (all other constructs) exponentials. Where bi-exponential fits were used, weighted *τ*_des_ is reported, calculated as follows: *τ*_w,des_ = *τ*_f_(*A*_f_/(*A*_f_ + *A*_s_)) + *τ*_s_(*A*_s_/(*A*_f_ + *A*_s_)), where *τ*_f(s)_ and *A*_f(s)_ represent the fast(slow) component time constant and coefficient, respectively.

### Synaptic recording

All procedures were carried out under PPL 70/8135 in accordance with UK Home Office regulations. Experiments conducted in the UK were licensed under the UK Animals (Scientific Procedures) Act of 1986 following local ethical approval. All animals were housed with food and water ad libitum on a 12 h light/dark cycle at room temperature (20–22 °C) and 45–65% humidity.

Organotypic slice cultures were prepared as previously described^74^. Briefly, hippocampi extracted from C57/Bl6 mice (postnatal day 6–8; either sex) were immersed in high-sucrose Gey’s balanced salt solution containing (in mM): 175 sucrose, 50 NaCl, 2.5 KCl, 0.85 NaH_2_PO_4_, 0.66 KH_2_PO_4_, 2.7 NaHCO_3_, 0.28 MgSO_4_, 2 MgCl_2_, 0.5 CaCl_2_ and 25 glucose at pH 7.3. Slices of 300 μm thickness were cut using a McIlwain tissue chopper and cultured on Millicell cell culture inserts (Millipore) in equilibrated slice culture medium (37 °C/5% CO_2_), containing 78.5% MEM, 15% heat-inactivated horse serum, 2% B27 supplement, 2.5% 1 M HEPES, 1.5% 0.2 M GlutaMAX supplement, 0.5% 0.05 M ascorbic acid, 1 mM CaCl_2_ and 1 mM MgSO_4_ (Thermo Fisher Scientific). Medium was exchanged every 3–4 d. Cultured slices were transfected (with GluA1 flip or GluA2 flip, Q/R-unedited, R/G-edited) via single-cell electroporation at 5–7 days in vitro, and recordings were obtained 4–6 d posttransfection.

Synaptic recordings were performed in aCSF solution containing (in mM): 10 glucose, 26.4 NaH_2_CO_3_, 126 NaCl, 1.25 NaH_2_PO_4_, 3 KCl, 4 MgSO_4_, 4 CaCl_2_ (to facilitate presynaptic glutamate release), 0.002 2-chloroadenosine, 0.1 D-AP5, 0.002 CGP52432 and 0.001 SR-95531, and saturated with 95% O_2_/5% CO_2_. Borosilicate pipettes were pulled to 3–6 MΩ and back-loaded with intracellular solution containing (in mM): 135 CH_3_SO_3_H, 135 CsOH, 4 NaCl, 2 MgCl_2_, 10 HEPES, 4 Na_2_-ATP, 0.4 Na-GTP, 0.15 spermine, 0.6 EGTA, 0.1 CaCl_2_, at pH 7.25. EPSCs were evoked by CA1 Schaffer collateral stimulation at 0.2 Hz using a monopolar glass electrode filled with aCSF, and responses were simultaneously recorded from a pair of GFP-positive and -negative cells situated in proximity. Whole-cell patch clamp signals were acquired using a Multiclamp 700B amplifier, digitized by Digidata 1550B (both Axon Instruments) and recorded using pCLAMP 10 (Molecular Devices). Rectification index was calculated from AMPAR currents measured in the whole-cell configuration at holding voltages of −60 mV, 0 mV and +40 mV as: rectification index = −(*I*_+40_ − *I*_0_)/(*I*_−60_ − *I*_0_). PPF was measured by normalizing averaged peak current amplitudes to the first EPSC in a train of 20 Hz stimulation. AMPAR-mediated PPF was recorded at −60 mV holding potential in the presence of 100 μM D-AP5, and NMDAR-mediated PPF was recorded at +40 mV holding potential with 10 μM NBQX^75^. No randomization or blinding was applied in these experiments.

### Reporting summary

Further information on research design is available in the Nature Portfolio Reporting Summary linked to this article.

## Online content

Any methods, additional references, Nature Portfolio reporting summaries, source data, extended data, supplementary information, acknowledgements, peer review information; details of author contributions and competing interests; and statements of data and code availability are available at 10.1038/s41586-023-06528-0.

### Supplementary information

Supplementary Fig. 1 Uncropped gel for Extended Data Fig. 1a.

Reporting Summary

Supplementary Video 1 Principal motions of resting GluA1/γ3 NTD. Structures are successively shown along each of three PCs of variation of NTD models, going to the two extremes of each motion then back to the average structure before going on to the next PC. A/C chains are light blue and B/D chains in dark blue. A reference LBD-TMD structure (PDB: 6QKZ) is overlaid and shown in transparent grey. PC1, PC2 and PC3 were scaled to maximum RMSDs of 20 Å, 9.85 Å and 6.78 Å, respectively, as described in Methods.

Supplementary Video 2 Non-swapped resting GluA1/γ3 structure rotating through different views. GluA1 A/C chains are colored light blue, B/D chains in dark blue and TARPs in two shades of brown.

Supplementary Video 3 Desensitized GluA1/γ3 structure with parallel NTDs and four-fold symmetric LBD rotating through different views. GluA1 A/C chains are colored light blue, B/D chains in dark blue and TARPs in two shades of brown.

Supplementary Video 4 Conformational changes in GluA1/γ3 desensitized LBD. A morph is shown for the LBD from the top, going from the reference A2-containing desensitized structure (PDB: 7QHH) through a series of GluA1/γ3 desensitized structures and back to the reference. Chains are colored as in other videos.

### Source data

Source Data Fig. 2

Source Data Fig. 3

Source Data Extended Data Fig. 6

Source Data Extended Data Fig. 8

Source Data Extended Data Fig. 10

## Figures and Tables

**Extended Data Table 1:**
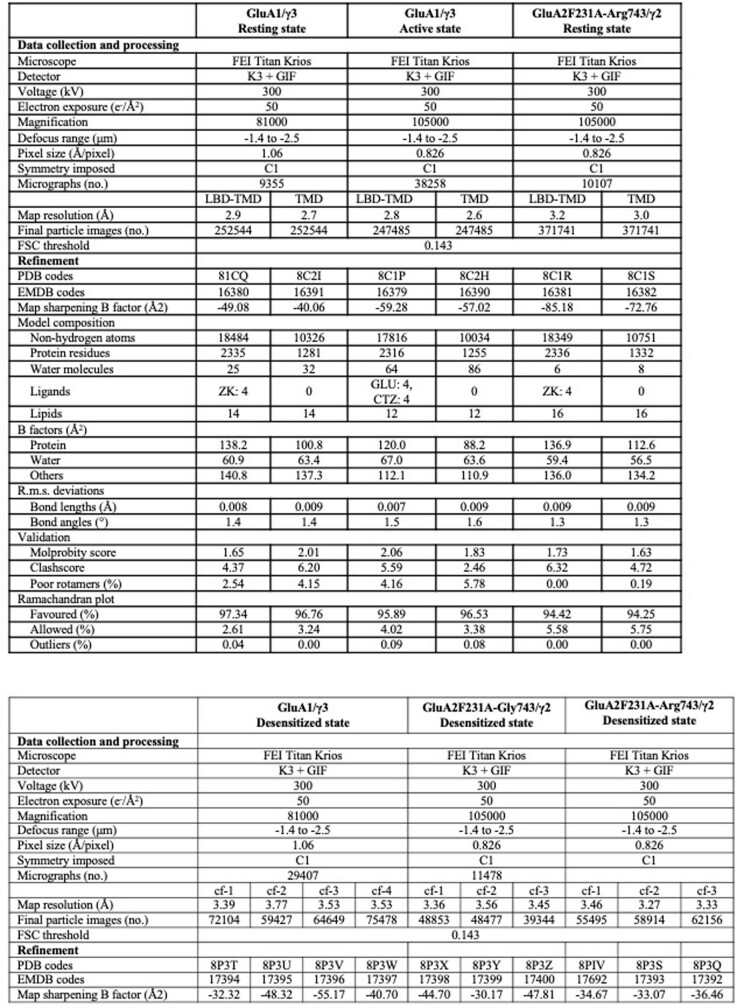
Cryo-EM data collection, refinement and validation statistics

**Extended Data Table 2:**
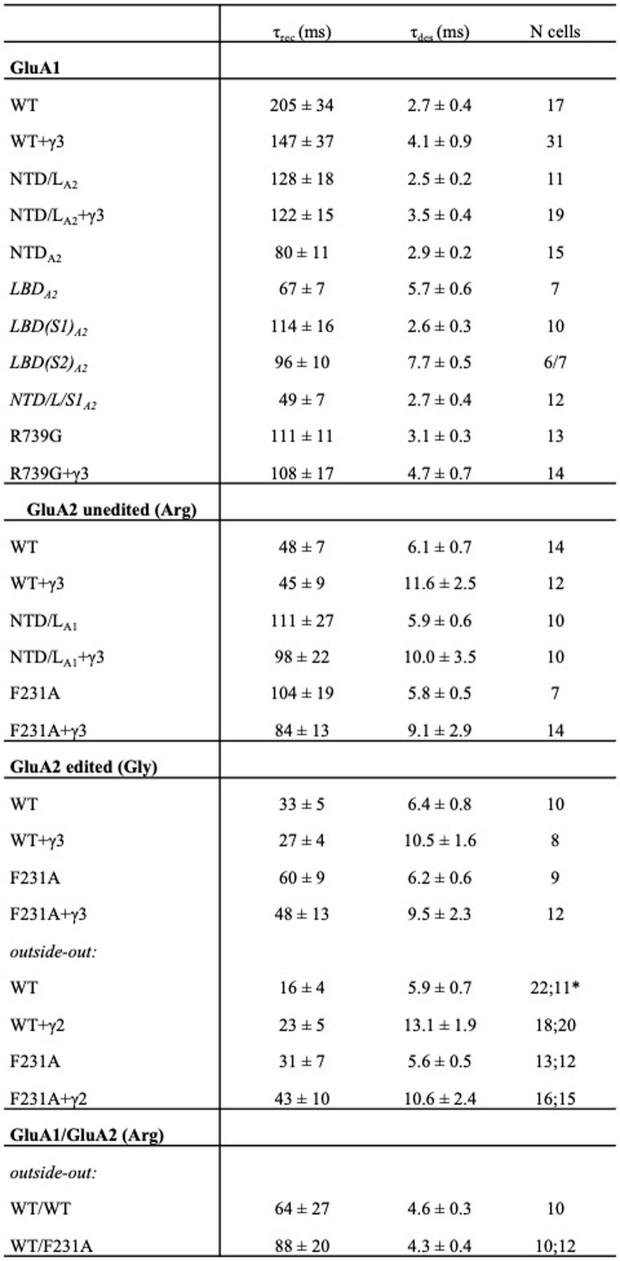
AMPAR recovery and desensitization parameters from whole cell and outside-out patches

^a^ for τ_rec_ and τ_rec_, respectively.

## Data Availability

Cryo-EM coordinates and corresponding EM maps are deposited in the PDB and EMDB under the accession codes: resting GluA1/γ3: 8C1Q/EMD-16380 (LBD–TMD) and 8C2I/EMD-16391 (TMD); active GluA1/γ3: 8C1P/EMD-16379 (LBD–TMD) and 8C2H/EMD-16390 (TMD); desensitized A1/γ3 LBD–TMD: 8P3T/EMD-17394 (cf-1), 8P3U/EMD-17395 (cf-2), 8P3V/EMD-17396 (cf-3) and 8P3W/EMD-17397 (cf-4); GluA2(F231A)/γ2 (Arg743) resting: 8C1R/EMD-16381 (LBD–TMD) and 8C1S/EMD-16382 (TMD); desensitized GluA2(F231A)/γ2 (Arg743): 8PIV/EMD-17692 (cf-1), 8P3S/EMD-17393 (cf-2), 8P3Q/EMD-17392 (cf-3); desensitized GluA2(F231A)/γ2 (Gly743): 8P3X/EMD-17398 (cf-1), 8P3Y/EMD-17399 (cf-2), 8P3Z/EMD-17400 (cf-3). Source data are provided with this paper.

## Code availability

ProDy is open source software that is available on GitHub (https://github.com/prody/ProDy), and through the Python package index (PyPI) with pip and the conda-forge channel for conda. Principal component analysis was performed using standard homogenous ensemble methods as described on the ProDy website.
