# Supplementary figures and images for: Structural mobility tunes signalling of the GluA1 AMPA glutamate receptor
Source: Nature. 2023 Sep 13;621(7980):877–82. doi: 10.1038/s41586-023-06528-0 (PMC10533411; doi:10.1038/s41586-023-06528-0)

## Slide 1
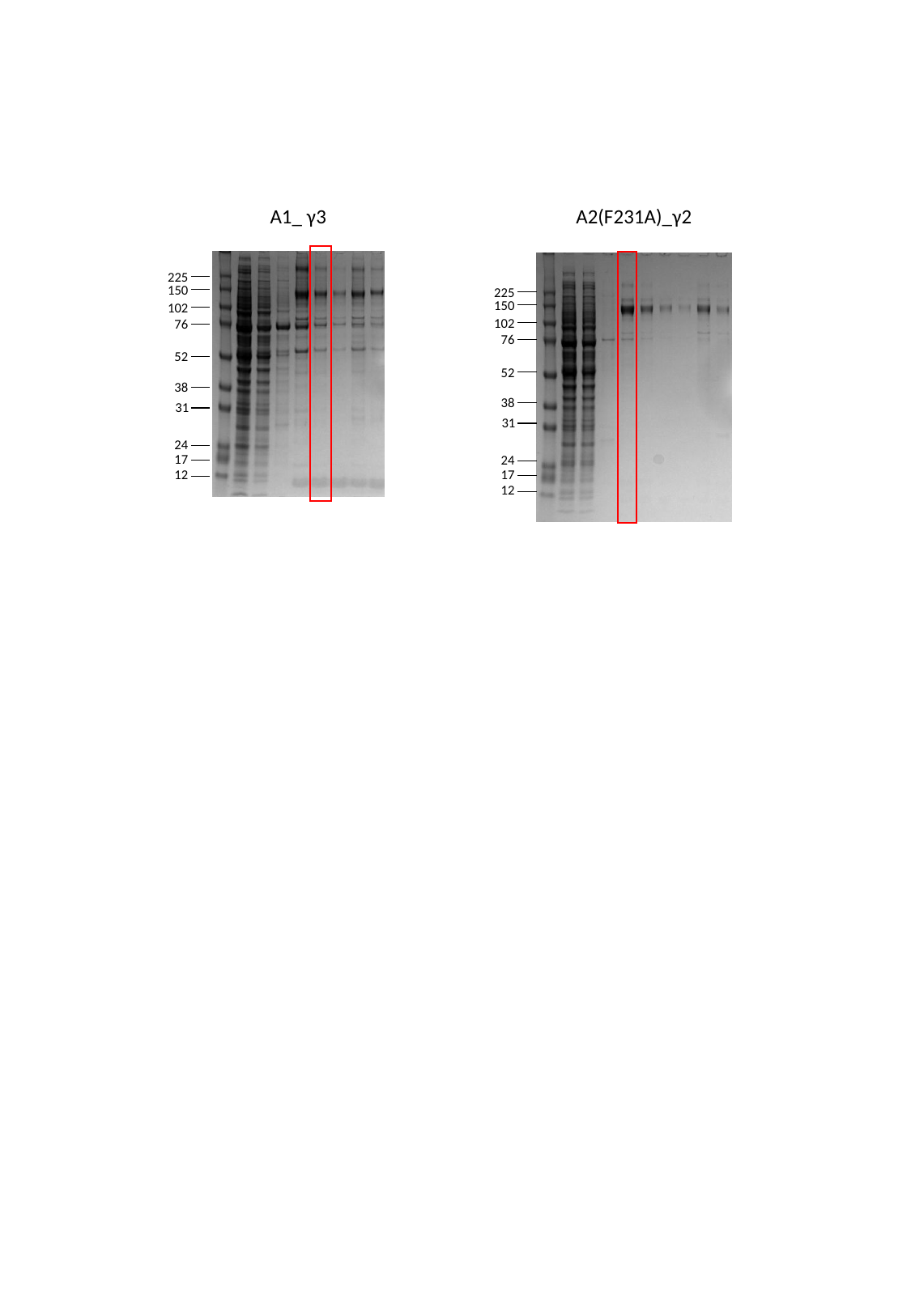

A2(F231A)_γ2
A1_ γ3
225
150
102
76
52
38
31
24
17
12
225
150
102
76
52
38
31
24
17
12

Supplement: Supplementary file 1 — Uncropped gel for Extended Data Fig. 1a. [file 41586_2023_6528_MOESM1_ESM.pptx]
